# Editorial: Letter of Recommendation

**DOI:** 10.1523/ENEURO.0357-16.2016

**Published:** 2016-12-15

**Authors:** Christophe Bernard

Dear friends and colleagues,

As you have already noticed, *eNeuro* is already a success: 100 papers published 15 months after launch, and more than 300, 9 months later! We are striving hard to make *eNeuro* the flagship of on-line open access neuroscience journals. Despite the reputation and appeal it has already gained, we fully understand that some authors may be hesitant to send their paper to a journal without an impact factor. This is the reason why, until we get our first impact factor (calculated in 2017, available in 2018), we have introduced the possibility for authors who have published a paper in *eNeuro* to request a letter of recommendation from The Society for Neuroscience. This letter will state that the published science achieves the highest standards of quality and will be signed by the Reviewing Editor of the paper, the Advisory Board member, and the Editor-in-Chief. Authors can include this letter in their CV to assist in their career progression. Please send any questions or comments to eNeuro@sfn.org.

